# A Common Susceptibility Gene for Type 2 Diabetes Is Associated with Drug Response to a DPP-4 Inhibitor: Pharmacogenomic Cohort in Okinawa Japan

**DOI:** 10.1371/journal.pone.0154821

**Published:** 2016-05-03

**Authors:** Uru Nezu Osada, Hiroshi Sunagawa, Yasuo Terauchi, Shinichiro Ueda

**Affiliations:** 1 Department of Clinical Pharmacology and Therapeutics, University of the Ryukyus Graduate School of Medicine, Okinawa Japan; 2 Department of Diabetes, Endocrinology and Metabolism, Saiseikai Yokohama City Nanbu Hospital, Yokohama, Japan; 3 Sunawaga Medical Clinic, Okinawa, Japan; 4 Department of Endocrinology and Metabolism, Yokohama City University Graduate School of Medicine, Yokohama, Japan; Children's National Medical Center, Washington, UNITED STATES

## Abstract

We investigated the association between common type 2 susceptibility variants of CDK5 regulatory subunit associated protein 1-like 1(*CDKAL1)* and therapeutic responses to anti-diabetic agents among patients with type 2 diabetes. Two SNPs (rs7754840: C>G, rs7756992: A>G) were genotyped via the Taqman PCR method. A total of 798 type 2 diabetic patients were included. HbA1c reduction after use of DPP-4 inhibitors for 3 months was significantly greater in patients with a risk allele for type 2 diabetes (GG -0.4%, CG -0.5%, CC -0.8%, p = 0.02 for rs7754840 and AA -0.4%, AG -0.5%, GG -0.8%, p = 0.01 for rs7756992). Linear regression analysis showed that per allele reductions of hemoglobin A1c (HbA1c) after 3 months were -0.10% for rs7754840 (p = 0.02) and -0.13% for rs7756992 (p = 0.0008) after adjusting for clinically influential covariates such as age, sex, BMI, duration of diabetes, baseline HbA1c and concomitant anti-diabetic agents. The results suggested that common variants of *CDKAL1* are associated with therapeutic response to DPP-4 inhibitors.

## Introduction

Inter-patient variability in responses to any drugs, including anti-diabetic agents (ADA), is large. Genetic factors may partly explain variability in drug responses because of altered pharmacokinetics or pharmacodynamics. For example, CYP2C9 is a sulfonylurea (SU) metabolic enzyme, and two common nonsynonymous variants (CYP2C9*2 and CYP2C9*3) are associated with decreased clearance and increased plasma concentration of SU[1][2], which might enhance therapeutic responses to SU [3].

CDK5 regulatory subunit associated protein 1-like 1 (*CDKAL1)* was recently identified as a susceptibility gene for type 2 diabetes (T2D) in genome-wide association studies [4–6]. A replication study showed that the C allele of rs7754840 and the G allele of rs7756992 were associated with increased risk of T2D in Japanese populations [4][5].

Although the function of *CDKAL1* is not yet clear, its protein product shares similarity with cyclin-dependent kinase 5 (*CDK5*), which encodes a protein primarily known to be an essential molecule in the brain. A recent *in vitro* study reported that CDK5 contributes to the impairment of pancreatic beta cell function [6]. In addition, pancreatic beta cells from mice lacking *CDKAL1* have a reduced first-phase insulin release *in vitro*; this altered insulin release may be due to blunted responsiveness of the K_ATP_ channel to glucose stimulation [7]. Actually, in an intravenous glucose tolerance test, carriers of the GC and CC genotypes of rs7754840 had insulin responses that were lower by 11 and 24%, respectively, than carriers of the GG genotype [8], suggesting that the C variant confers T2D risk through reduced insulin secretion.

We hypothesized that *CDKAL1* variants might affect therapeutic responses to ADA, especially to insulin stimulators. Therefore, we conducted a genome cohort study of T2D patients in Okinawa, Japan.

## Materials and Methods

### Study design

This is a retrospectively registered, historical cohort study.

### Patients

T2D patients who commenced treatment with any of the following classes of ADA sometime between April 1, 2006 (clinic inception) to June 18, 2012 were included: 1) Biganides (BG), 2) SU, 3) Dipeptidyl peptidase 4 inhibitors (DPP-4I), 4) Thiazolidinediones (TZD), 5) Glinides (GLN), and 6) Glucagon-like peptide 1 (GLP1). We used an automated retrieval system of electronic medical record to identify candidate patients. We then manually reviewed all the medical records in detail and excluded patients with any of the following: 1) type 1 diabetes mellitus, 2) monogenic disorder known to cause diabetes, 3) absence of a clinic visit from January 1st to June 18th, 2012, 4) receipt of ADA continuously at referral hospital before registration.

### Informed consent and ethical considerations

During the period for enrolment of patients (from August 1, 2012 to July 31, 2013), we attempted to obtain written informed consent from each eligible patient. If a patient had a compromised capacity to consent because of dementia or mental retardation, informed consent was obtained from their legal guardian or next of kin. Patients were informed of their right to withdraw from the study without compromising their treatment. The committee for ethical research at University of the Ryukyus approved the study protocol on the date of June 20, 2012 (approval number 61).

### Sample collection and preparation of genomic DNA

After obtaining written informed consent, we collected 5 ml of peripheral venous blood into tubes containing EDTA-Na_2_. After labeling each sample with a study number for patient anonymity, we transported the samples to Ryukyu University and stored them at -30°C until DNA extraction. QIAamp DNA Mini Kits (Qiagen K.K., Tokyo) were used according to the manufacturer’s instructions to extract genomic DNA was extracted from the peripheral leukocytes fraction; each DNA sample was stored at -30°C until genotyping.

### Genotyping

We used TaqMan^®^ SNP genotyping assays (TaqMan Validated SNP assays C_29246232_10 and C_2504058_20, Applied Biosystems, Foster City, CA) to genotype two SNPs, rs7754840 (G > C) and rs756992 (A > G) in each sample. The PCR amplification was done in duplicated wells with single negative control that contain everything but the template DNA in order to eliminate contamination. The assay volume was 25 μl; each reaction contained 13.25 μl of reaction mix (TaqMan Universal PCR Master Mix No AmpErase UNG, SNP genotyping assay including two Taqman MGB probes labeled with FAM and VIC fluorescence dyes) and 11.75 μl of DNA (1.7 ng/μl). The universal temperature cycling included polymerase activation at 95°C for 10 min, followed by 40 cycles of denaturation at 92°C for 15 s and annealing/extension/plate reading at 60°C for 1 min. PCR amplification was done with a ABI PRISM 7000 system and StepOnePlus^TM^ (Applied Biosystems). Genotypes were automatically determined by the corresponding software (ABI PRISM 7000 Sequence Detection System software v 1.2.3 and StepOne^TM^ software v 2.2.2). Any ambiguous genotype was reanalyzed.

### Clinical endpoints, data management, and statistical analysis

The clinical data and the genotyping results were stored and managed at University of the Ryukyus. The primary endpoints were change in plasma glucose (PG) and change HbA1c at 3 months after start of a new ADA prescription. The secondary endpoint was change in HbA1c at other time points (1, 6, 12 months). Associations between genotypes and glucose-lowering effects of each ADA, as represented by a reduction in PG/HbA1c, were tested in all eligible patients. For sensitivity analyses, the association between effects of ADA and genotypes was tested in patients who were taking ADA continuously throughout the observation period. We further excluded all data from a patient if there was 1) a new prescription, 2) discontinuation, or 3) a dosage change of any other ADA within 3 months prior to start of the ADA of interest.

Lastly, we used linear regression analysis to estimate the HbA1c reduction expected per one T2D risk allele in each SNPs. We adjusted for each of the following six potentially confounding clinical characteristics: age, gender, BMI, HbA1c, diabetic duration and concomitant ADA.

The results for continuous variables are presented as mean value ± standard deviation (SD), 95% CI or medians with interquartile ranges; categorical variables are presented as number and ratio (%). Since we evaluated two SNPs, the significance level was set at 2.5%. We calculated linkage disequilibrium (LD) coefficients (*r*^2^) according to the method by Delvin et al [9]. The deviations from Hardy-Weinberg equilibrium (HWE) of allele and genotype frequencies for the SNPs were evaluated by Pearson's χ^2^ analysis with a χ^2^ value of ≤ 3.84 indicating significance. We assessed the differences between the groups with analysis of variance (ANOVA) and *t* test for continuous variables and with the χ^2^ test for categorical variables. We conducted all statistical analyses using JMP 11 (SAS institute Inc).

### Blinding

SNP results were kept undisclosed to both patients and physicians until the end of the observation period.

## Results

After a database search of electronic medical records, we initially identified 1494 candidate patients who had started the relevant ADA between April 1st, 2006 and June 18^th^ 2012. After reviewing medical records, we excluded 600 patients; 1) 437 with no clinic visit from January 1^st^ to June 18^th^ 2012 and 2) 163 whose ADA prescription was started at a referral hospital. Among the 894 eligible patients, we could not obtain informed consent from 96 patients; 1) 44 did not visit the clinic during the entry period, 2) 22 did not consent, 3) 15 were transferred to another hospital, 4) 7 exhibited cognitive dysfunction, 5) 4 were excluded based on a physician’s decision and 6) 4 withdrew consent. Ultimately, 798 patients participated in the study.

### Baseline Characteristics

Table 1 shows the patients' baseline characteristics. Among the 798 participants, mean age was 63.2 years and 49% were male. On average, patients were obese (BMI 27 ± 4.8 kg/m2) and their duration of diabetes was for 10 years (IQR 5–16). Baseline PG/HbA1c was 208.2 ± 83.4 mg/dl and 8.6 ± 1.7%.

**Table 1:** Baseline characteristics of patients.

Variable	Value	Variable	Value
N (%)	798	Comorbidities, n (%)	
Age (year)	63.2 ± 12.0	Hypertention	567 (71)
Male, n (%)	389 (49)	Dislipidemia	660 (83)
Height (cm)	157 ± 9.1	Coronary artery disease	82 (10)
BMI (kg/m^2^)	27.0 ± 4.8	Cerebrovascular disease	64 (8)
sBP (mmHg)	131 ± 18	Lacuna infarction	32 (4)
dBP (mmHg)	71 ± 12	Transient ischemic attack	9 (1)
Heart rate (/min)	78 ± 10	Peripheral arterial disease	24 (3)
Plasma glucose (mg/dl)	208.2 ± 83.4	Atrial fibrillaltion	17 (2)
HbA1c (%)	8.6 ± 1.7	Cancer	42 (5)
eGFR	90.8 (69.6–117.9)	Diabetic retinopathy	
Cholesterol (mg/dl)	200.3 (42.5)	No	413 (52)
LDL cholesterol (mg/dl)	113 (37.6)	Yes	234 (29)
HDL cholesterol (mg/dl)	53.7 (14.1)	Simple	127 (16)
Triglycerides (mg/dl)	153 (107–208)	Preproliferative	58 (7)
Smoking status, n (%)		Proliferative	49 (6)
Never	453 (60)	Non-diabetic retinal disease	2 (0.3)
Current	116 (15)	Unknown	149 (19)
Past	188 (25)	Diabetic nephropathy	
Unknown	41 (5)	Stage 1	445 (56)
Alcohol use, n (%)		Stage 2	198 (25)
Never	477 (60)	Stage 3	97 (12)
Yes	269 (34)	Stage 4	25 (3)
1–3 day/week	159 (20)	Stage 5	21 (3)
4–7 day/week	110 (14)	Non-diabetic kidney disease	11 (1)
Unknown	52 (7)	Unknown	149 (19)
Duration of diabetes (year)	10 (5–16)		
Diabetic family history, n (%)			
None	319 (40)		
Yes	433 (54)		
1st degree family	305 (38)		
2nd degree family	107 (13)		
3rd degree family	21 (3)		
Unknown	45 (6)		

Stage 1; albuminuria < 30 mg/24 h, Stage 2; albuminuria 30–299 mg/24 h, Stage 3; albuminuria > 300 mg/24 h, Stage 4; Cre > 1.3 mg/dl, Stage 5; patients receiving dialysis

We obtained blood samples from 786 patients and purified genomic DNA from each sample. Taqman SNP genotyping revealed the genotype distribution for *CDKAL1* rs7754840 SNP was GG 34%, GC 46%, and CC 20%; that of rs7756992 was AA 28%, AG 48%, and GG 24%. The minor allele frequencies of rs7754840 and rs7756992 were 43.0% and 48.1%, respectively. A considerable linkage disequilibrium existed between the two SNPs (D' = 0.979, r2 = 0.7805), which was consistent with both Japanese (r^2^ = 0.745)[10] and Caucasian (r^2^ = 0.677) [8] in Hapmap database. Hardy-Weinberg equilibrium was confirmed by χ^2^ square test, in which χ^2^ was 2.76 for rs7754840 and 0.73 for rs7756992 (< 3.84, the significant threshold of p-value 0.05). Baseline characteristics did not differ between genotypes for either SNP.

One patient was treated by three ADAs in average. Thus the total number of target of analysis was 1621 (n = 522 for BG, n = 252 for SU, n = 512 for DPP-4I, n = 276 for TZD, n = 28 for GLN, n = 31 for GLP1).

### Therapeutic response according to *CDKAL1* genotype

**Fig 1:**
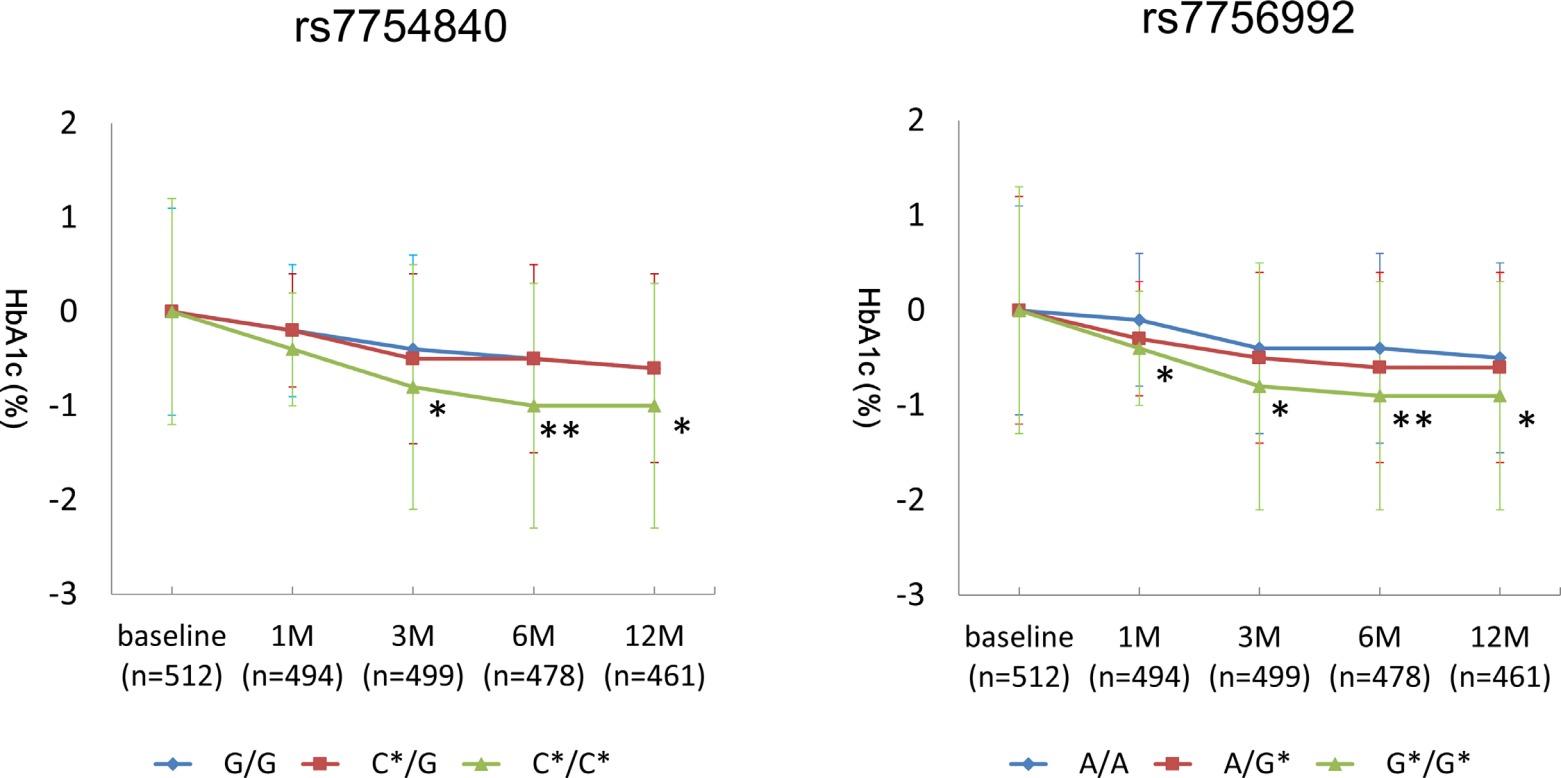
Change in HbA1c after DPP4-I treatment according to *CDKAL1* genotypes. SNP rs7754840 (left) and rs7756992 (right). Each line shows a change in HbA1c from baseline.* P < 0.025 and **P < 0.001 for comparisons between genotypes.

Overall, PG/HbA1c was significantly improved by each ADA (S1 Table and Fig 1). There were no significant differences in response of PG/HbA1c to BG, SU, GLN, TZD, or GLP1 between genotypes. However as for DPP-4I, HbA1c reduction was significantly greater in patients who carried a T2D risk allele (GG -0.4%, CG -0.5%, CC -0.8%, p = 0.02 for rs7754840 and AA -0.4%, AG -0.5%, GG -0.8%, p = 0.01 for rs7756992).

Several sensitivity analyses were performed. First, those patients with any of the following were excluded: 1) a new prescription, 2) discontinuation, or 3) dosage changes of any other ADA within 3 months prior to the DPP-4I prescription. As the result (Table 2), although HbA1c reduction at 3 months did not differ among genotypes; HbA1c reduction at 6 months was greater in patients with a T2D risk allele (GG -0.6%, CG -0.8%, CC-1.2% for rs7754840, p = 0.07 and AA -0.6%, AG -0.8%, GG -1.2%; p = 0.03 for rs7756992). In head-to-head comparisons between each pair of homozygous genotypes, the differences were more prominent (GG vs CC, p = 0.04 for rs7754840 and AA vs GG, p = 0.02 for rs7756992, data not shown).

**Table 2:** HbA1c after DPP4-I in patients with no prior prescription, discontinuation or dosage change of ADA other than DPP-4I.

**rs7754840**	**GG (n = 59)**	**CG (n = 78)**	**CC (n = 38)**	**P**
Baseline (n = 177)	7.8 ± 1.1	7.9 ± 1.2	7.8 ± 1.0	0.93
1 month (n = 168)	-0.3 ± 0.4	-0.3 ± 0.7	-0.5 ± 0.6	0.27
3 months (n = 166)	-0.7 ± 0.9	-0.7 ± 1.0	-0.9 ± 0.9	0.5
6 months (n = 156)	-0.7 ± 0.9	-0.8 ± 0.9	-1.2 ± 0.9	0.07
12 months (n = 147)	-0.8 ± 0.9	-0.8 ± 1.0	-1.1 ± 1.1	0.32
**rs7756992**	**AA (n = 46)**	**AG (n = 84)**	**GG (n = 45)**	**P**
Baseline (n = 177)	7.9 ± 1.0	7.9 ± 1.2	7.8 ± 1.0	0.98
1 month (n = 168)	-0.3 ± 0.3	-0.3 ± 0.6	-0.3 ± 0.6	0.18
3 months (n = 166)	-0.7 ± 0.8	-0.6 ± 1.0	-0.9 ± 0.9	0.24
6 months (n = 156)	-0.6 ± 0.9	-0.8 ± 0.9	-1.2 ± 1.0	0.03
12 months (n = 147)	-0.7 ± 0.8	-0.8 ± 1.0	-1.0 ± 1.1	0.35

Values are mean ± SD. P values are based on comparison between genotypes (ANOVA)

Next, we extracted the only data with no DPP-4I discontinuation after initiation (Table 3). HbA1c reduction at 3 months was still greater in patients with a T2D risk allele (n = 479, GG -0.5%; CG -0.5%; CC -0.8%; p = 0.02 for rs7754840 and AA -0.4%; AG -0.5%; GG -0.8%; p = 0.01 for rs7756992). The differences were even more prominent at 6 and 12 months.

**Table 3:** HbA1c after DPP-4I in patients with no drug discontinuation.

**rs7754840**	**GG (n = 175)**	**CG (n = 237)**	**CC (n = 100)**	**P**
Baseline (n = 512)	7.8 ± 1.1	7.8 ± 1.2	7.9 ± 1.3	0.73
1 month (n = 491)	-0.2 ± 0.7	-0.2 ± 0.6	-0.4 ± 0.6	0.03
3 months (n = 479)	-0.5 ± 0.9	-0.5 ± 0.9	-0.8 ± 1.3	0.02
6 months (n = 452)	-0.5 ± 1.0	-0.5 ± 1.0	-1.0 ± 1.3	0.0003
12 months (n = 425)	-0.6 ± 1.0	-0.7 ± 1.0	-1.0 ± 1.3	0.01
				
**rs7756992**	**AA (n = 46)**	**AG (n = 84)**	**GG (n = 45)**	**P**
Baseline (n = 512)	7.8 ± 1.1	7.8 ± 1.2	7.9 ± 1.3	0.76
1 month (n = 491)	-0.1 ± 0.7	-0.3 ± 0.6	-0.4 ± 0.6	0.01
3 months (n = 479)	-0.4 ± 0.9	-0.5 ± 0.9	-0.8 ± 1.3	0.01
6 months (n = 452)	-0.4 ± 1.1	-0.6 ± 1.0	-1.0 ± 1.3	0.0004
12 months (n = 425)	-0.5 ± 1.0	-0.7 ± 1.0	-1.0 ± 1.3	0.004

Values are mean ± SD. P values are based on comparison between genotypes (ANOVA)

Furthermore, we divided the cases into DPP-4I monotherapy or combination therapy (Table 4). The majority of patients (n = 426, 83%) were in combination therapy with another ADA. DPP-4I monotherapy was used by only 86 patients (17%). HbA1c reduction was numerically better in patients who were homozygous for a T2D risk allele, but this difference was not statistically significant.

**Table 4:** HbA1c after DPP-4I monotherapy or combination therapy.

	rs7754840				rs7756992			
**Monotherapy (n = 86)**	**GG (n = 27)**	**CG (n = 38)**	**CC (n = 21)**	**P**	**AA (n = 22)**	**AG (n = 40)**	**GG (n = 24)**	**P**
Baseline	7.4 ± 1.0	7.1 ± 1.0	7.2 ± 1.0	0.69	7.4 ± 0.2	7.1 ± 0.2	7.2 ± 0.2	0.55
1months	-0.2 ± 0.7	-0.2 ± 0.7	-0.4 ± 0.7	0.61	-0.2 ± 0.70	-0.13 ± 0.70	-0.38 ± 0.70	0.53
3months	-0.4 ± 1.0	-0.4 ± 1.0	-0.6 ± 1.0	0.64	-0.5 ± 0.9	-0.3 ± 0.9	-0.7 ± 0.9	0.29
6months	-0.5 ± 0.9	-0.6 ± 0.9	-0.9 ± 0.9	0.38	-0.4 ± 0.9	-0.5 ± 0.9	-0.9 ± 0.9	0.13
12months	-0.5 ± 1.0	-0.5 ± 1.0	-0.8 ± 1.0	0.35	-0.5 ± 1.0	-0.4 ± 1.0	-1.0 ± 1.0	0.09
**Combination (n = 426)**	**GG (n = 149)**	**CG (n = 197)**	**CC (n = 80)**	**P**	**AA (n = 114)**	**AG (n = 215)**	**GG (n = 97)**	**P**
Baseline	7.9 ± 1.2	7.9 ± 1.2	8.1 ± 1.2	0.38	7.9 ± 1.2	7.9 ± 1.2	8.0 ± 0.2	0.53
1months	-0.2 ± 0.6	-0.2 ± 0.6	-0.4 ± 0.6	0.04	-0.1 ± 0.6	-0.3 ± 0.6	-0.4 ± 0.6	0.006
3months	-0.4 ± 1.0	-0.5 ± 1.0	-0.8 ± 1.0	0.02	-0.3 ± 1.0	-0.5 ± 1.0	-0.8 ± 1.0	0.01
6months	-0.5 ± 1.1	-0.5 ± 1.1	-1.1 **±** 1.1	4E-04	-0.4 ± 1.1	-0.6 ± 1.1	-1.0 ± 1.1	0.001
12months	-0.6 ± 1.1	-0.6 ± 1.1	-1.0 ± 1.1	0.02	-0.5 ± 1.1	-0.7 ± 1.1	-0.9 ± 1.1	0.009

Values are mean ± SD. P values are based on comparison between genotypes (ANOVA)

### Multivariate analysis of HbA1c after DPP-4I

In order to eliminate confounding effect, we performed multivariate analysis by linear regression analysis (Table 5). As the covariates, we adjusted age, sex, BMI, diabetes duration, HbA1c, and the number of ADAs that were concomitantly used with DPP4-I at baseline. Reduction of HbA1c after 3 months was -0.10% per rs7754840 risk allele (p = 0.02) which was statistically significant, and the significant reduction was maintained after 12 months. Similarly for rs7756992, per risk allele reduction of HbA1c was also significant throughout the whole observation period (HbA1c -0.08 to -0.17).

**Table 5:** Multivariate analysis of HbA1c after DPP-4I.

SNP	Covariates	1 months		3 month		6 month		12 month	
		β	P	β	P	β	P	β	P
rs7754840	T2D risk allele (n)	-0.07	0.04	-0.1	0.02	-0.13	0.01	-0.13	0.01
	Age (year)	0.00007	0.97	-4E-04	0.9	-0.004	0.32	0.003	0.32
	Female	0.005	0.85	0.05	0.15	0.07	0.08	0.05	0.15
	Duration of diabetes (year)	0.004	0.18	0.008	0.05	0.009	0.04	0.003	0.49
	BMI (kg/m^2^)	0.001	0.77	0.009	0.26	0.005	0.54	0.001	0.89
	HbA1c (%)	-0.24	<0.0001	-0.54	<0.0001	-0.59	<0.0001	-0.58	<0.0001
	Concomitant ADA (n)	0.06	0.01	0.13	0.0001	0.2	<0.0001	0.19	<0.0001
rs7756992	T2D risk allele (n)	-0.08	0.02	-0.13	0.008	-0.18	0.001	-0.17	0.002
	Age (year)	-5E-05	0.98	-6E-04	0.85	-0.003	0.28	0.003	0.36
	Female	0.003	0.89	0.05	0.16	0.07	0.08	0.05	0.17
	Duration of diabetes (year)	0.004	0.18	0.008	0.05	0.009	0.04	0.003	0.48
	BMI (kg/m^2^)	0.002	0.76	0.008	0.27	0.005	0.55	0.0009	0.9
	HbA1c (%)	-0.24	<0.0001	-0.54	<0.0001	-0.58	<0.0001	-0.58	<0.0001
	Concomitant ADA (n)	0.05	0.02	0.13	0.0002	0.19	<0.0001	0.18	<0.0001

P-values were calculated by multiple linear regression analysis. β- Coefficient, SE: standard error

## Discussion

This was a pragmatically designed genome cohort study intended to assess the association between therapeutic response to ADA and T2D susceptibility alleles. Our analysis showed that common T2D risk variants of *CDKAL1* showed better HbA1c reduction with DPP-4I.

It is not easy to know the exact therapeutic response to a single ADA in a T2D patient. In the first, the dosage of the ADA is not always same and might affect the therapeutic response. However, those information were not investigated in our database thus could not be analyzed. In the next, T2D patients are often treated with multiple ADAs. In our study patients, more than 80% of patients using DPP-4I used it in combination with another ADA. In cases involving DPP4-I monotherapy in Table 4, the intergroup difference in HbA1c response was not statistically significant; however, this lack of significance was probably due to the small sample size (n = 86, 17%).

The GLP1 agonist is another incretin agent; therefore, it may also potentiate SNP-response association in a manner similar to DPP-4I. In our study, no ADA besides DPP-4I showed significant association with these genetic variants. However, most of the analyses were underpowered due to the small sample size. Sample size involving GLP1 cases was only 31, which was too small to conclude a negative result.

### Comparison with other studies

To our knowledge, there are two PGx studies of ADA that investigated *CDKAL1* variants [11][12]. Chistiakov et al. showed a lack of association between responses to DPP4-I and *CDKAL1* variants in a Russian population; however, this result might be due to relatively small sample size (n = 69) compared to ours (n = 512) (13). They reported the post-meal excursion of endogenous insulin after SU and that of PG after GLN were better in patients with T2D risk genotypes at *CDKAL1* SNPs (rs10946398, rs7754840 and rs7756992) [11]. Similarly, Schroner et al. recently reported that fasting PG after SU was more reduced in patients with GG genotype at rs7756992 [12]. In terms of association between PG and *CDKAL1* SNPs, we could not replicate their results regarding SU/GLN partly because accurate measurement of fasting glucose was difficult in our study setting and the number of patients receiving GLN in our cohort was not large enough to detect possible association. However, even if *CDKAL1* SNPs affect fasting or post-prandial glucose after SU or GLN in experimental study setting, they might not affect overall glucose control represented by HbA1c. It should be confirmed in another prospective study with a larger sample size.

There is one PGx study of DPP-4I,which reports that rs7202877 SNP near CTRB1/2 was associated with lower HbA1c response to DPP-4I in type 2 diabetes [13]. This SNP on chromosome 16q23.1 has been identified as a risk factor for type 1 diabetes [14] and as a protective factor for type 2 diabetes [15], and increase gene expression of CTRB1/2 in islets and pancreata and enzyme activity in stool, implicating a key role of the enteroendocrine system. However, it is not unknown whether there is a link between the role between *CDKAL1* and CTRB1/2.

### Mechanism

The mechanism underlying the association between *CDKAL1* and the therapeutic response is not clear. There are several studies that indicate a pathogenic role of *CDKAL1* in pancreatic beta cells. Ohara-Imaizumi et al. reported that *CDKAL1* knockout mice showed reduced first-phase insulin exocytosis and impairment of mitochondrial ATP generation [7]. Mitochondrial ATP generation is a key step for insulin exocytosis in two different pathways. First, ATP is a facilitating factor of a KATP channel and it later induces Ca^2+^ influx by activating a Ca^2+^ channel, which is called a triggering pathway [16]. This may explain the association between *CDKAL1* variants and therapeutic response to SU/GLN that was reported previously [11][12]. KATP channels are hetero-octamers assembled from potassium inward rectifier 6.2 subunit (Kir6.2) and the SU receptor 1 (SUR1) and thus play an essential role in the pharmacological effect of SU/GLN [17]. Secondly, mitochondria-generated ATP has direct effects on insulin exocytosis at steps distal to the triggering pathway which is regarded as a part of amplifying pathway [16]. Both of these pathways may be hampered in carriers of *CDKAL1* risk variants, in which actually impaired insulin secretion is reported [18][19][8][20]. DPP-4I is known to stimulate insulin secretion through intracellular cAMP signaling and subsequent Ca^2+^ influx in pancreatic beta cells; this route is another part of the amplifying pathway [16]. DPP-4I might have substituted the suppressed ATP level in carriers of *CDKAL1* risk variants, leading to better clinical response. More mechanistic research is needed to elucidate the pharmacophysiology of *CDKAL1*.

### Strength and Limitations

As a strength of our the study, the minor allele frequencies were in accordance with the previous observation in Japanese populations [4] [21]. Additionally, although the information bias is generally a major concern in observational studies, we kept the SNP results undisclosed to both the patients and the physicians until the study end. Therefore, the clinical outcomes could not be influenced by the information bias.

There are several limitations in this study. In the first, although the difference in therapeutic response was statistically significant, it is not equal to the clinical significance. The difference in HbA1c per risk allele was smaller than 0.2% after one year treatment, which is not enough to change clinician’s attitude toward ADA prescription. In the next, the information bias is generally of concern with an observational study design. However, the data reflect the real practice of targeting a broad spectrum of patients. In addition, the sensitivity analyses seeking for true association showed consistent results. Lastly and the most importantly, our study result is not still applicable for clinical practice. The reason is that 1) non-PGx strategy of the drug optimization has been working well in general, and 2) it is not clear whether drug optimization aiming for lowering HbA1c improves mortality and morbidity in the future. In order to promote PGx in T2D treatment, we also need to verify its cost-effectiveness in comparison with conventional strategy using rigorous method such as random control trials.

## Conclusion

In summary, common variants of *CDKAL1* (rs7754840 and rs7756992) were associated with therapeutic response to DPP-4I in patients with T2D.

## Supporting Information

S1 Table Therapeutic response according to CDKAL1 genotype. Values are mean ± SD. P values are based on comparison between genotypes.
(DOCX)
