# Supplementary material for: A Common Susceptibility Gene for Type 2 Diabetes Is Associated with Drug Response to a DPP-4 Inhibitor: Pharmacogenomic Cohort in Okinawa Japan
Source: PLoS One. 2016 May 3;11(5):e0154821. doi: 10.1371/journal.pone.0154821 (PMC4854407; doi:10.1371/journal.pone.0154821)
Supplement: S1 Table — Values are mean ± SD. P values are based on comparison between genotypes. (DOCX) [file pone.0154821.s001.docx]

S1 Table―Therapeutic response according to CDKAL1 genotype

Values are mean ± SD. P values are based on comparison between genotypes.

S1 Table―Therapeutic response according to CDKAL1 genotype―*Continued*

**

Values are mean ± SD. P values are based on comparison between genotypes.
